# The cognitive and behavioral effects of *D*-amphetamine and nicotine sensitization in adult zebrafish

**DOI:** 10.1007/s00213-021-05844-5

**Published:** 2021-05-08

**Authors:** Madeleine Cleal, Barbara D. Fontana, Matthew O. Parker

**Affiliations:** School of Pharmacy and Biomedical Science, University of Portsmouth, St Michael’s Building, White Swan Road, Portsmouth, PO1 2DT UK

**Keywords:** FMP Y-maze, Zebrafish, Behavioral sensitization, Amphetamine, Nicotine, Addiction

## Abstract

**Background:**

Zebrafish are growing in use as a model for understanding drug dependence and addiction. Sensitization paradigms have been a useful tool in identifying mechanisms involved in drug-induced behavioral and neurological changes, but in zebrafish have tended to focus on locomotor, rather than cognitive, endpoints.

**Methods:**

Here, we used a novel method, the FMP Y-maze, which measures continuous performance through a series of repeated binary choices (L vs R), to establish a model for assessing parameters associated with psychostimulant-induced behavioral and cognitive sensitization in adult zebrafish.

**Results:**

Repeat, intermittent exposure to *d*-amphetamine (AMPH) for 14 days increased alternations (LRLR) in the maze, suggesting improved working memory, which was enhanced further following drug challenge after a short withdrawal period, suggesting behavioral sensitization. However, this cognitive enhancement coincided with a reduction in the use of other exploration strategies, hypolocomotion, and inhibition of cognitive flexibility. Like AMPH, exposure to nicotine (NIC) increased alternations following drug challenge after chronic treatment. Repeat NIC exposure appeared to induce both cognitive and psychomotor sensitization, as evidenced by increased working memory performance (alternations) and locomotor activity, without negatively impacting other search strategies or cognitive flexibility.

**Conclusion:**

Chronic treatment with AMPH or NIC boosts cognitive performance in adult zebrafish. Cognitive sensitization occurred with both drugs, resulting in enhanced working memory; however, repeat AMPH exposure, following a withdrawal period, resulted in inhibited cognitive flexibility, an effect not evident with repeat NIC exposure. Cognitive and behavioral sensitization paradigms in zebrafish could serve as a useful tool for assessing cognitive states which result in cognitive enhancing or impairing effects of drugs.

## Introduction

Psychostimulants, such as amphetamine (AMPH), nicotine (NIC), and cocaine, are known to increase extracellular synaptic dopamine (DA) concentrations and activate DA neurotransmission, resulting in altered behavioral and locomotor responses (Brown and Kolb 2001; Cunningham et al. 1997; Dela Peña et al. 2015; Di Chiara and Bassareo 2007; Niculescu et al. 2005; Volkow et al. 1999). Repeated administration of a psychostimulant drug enhances psychomotor responsiveness to the drug. This is mediated by enhancement of dopaminergic activation and modification of dendritic morphology, which can last for extended periods (Robinson and Berridge 1993). This long-term ability of previously encountered drugs to activate DA neurotransmission and modify behavioral outputs is known as drug-induced sensitization. Sensitization to repeated, intermittent drug exposure in humans is theorized to be a critical driver in maintaining drug use and escalation from casual experimentation to craving and abuse. Furthermore, those attempting to withdraw from their habit can relapse following long periods of abstinence (Robinson and Berridge 2008; Vezina 2007). The psychostimulants AMPH and NIC have been well documented for modulating locomotor responses (Buenrostro-Jáuregui et al. 2016; Cunningham et al. 1997; Pisera-Fuster et al. 2019; Valjent et al. 2010), as well as cognitive altering effects in both animals and humans (Kuhn et al. 2019; Steketee and Kalivas 2011; Vezina 2007; Vezina and Leyton 2009). Expanding our understanding of the mechanisms underlying drug-induced sensitization may boost the efficiency of therapies used to treat substance abuse and reduce the probability of relapse.

Conservation of the ascending midbrain dopaminergic pathways between mammals and teleost fish, such as zebrafish, has resulted in the increasing use of zebrafish as a neurobehavioral model for assessing the effects of pharmaceutical and illicit drugs (Antunes and Biala 2012; Barros et al. 2008; Goldsmith 2004; Kalueff et al. 2014). Behavioral responses of zebrafish to psychostimulant drug exposure (e.g. amphetamine (Parker et al. 2012), cocaine (Lopez Patino et al. 2008), or nicotine (Suen et al. 2013)) have corroborated findings in rodents and humans, thus supporting the suitability of zebrafish as a translational model of psychostimulant abuse (Parker and Brennan 2012; Stewart et al. 2015). There have been several studies that have demonstrated drug-induced sensitization in zebrafish (Blaser et al. 2010; Pisera-Fuster et al. 2019). Although these studies have been a useful starting point, they have yet to further our understanding of the mechanisms underpinning sensitization, and the relationship between sensitization, addiction, and relapse. In addition, despite extensive investigation into psychomotor sensitization, few studies have investigated the role of cognitive sensitization (Castner and Goldman-Rakic 2004; Muhammad and Kolb 2011; Peleg-Raibstein et al. 2009) and none, to our knowledge, has done so in zebrafish.

Here, we aimed to establish whether young-adult zebrafish develop cognitive or psychomotor sensitization to repeated administrations of AMPH and NIC, as assessed through the impact on working memory, locomotor activity, and cognitive flexibility. To do this, we used an established behavioral assay for zebrafish that has high translational relevance to humans, the FMP Y-maze (Cleal et al. 2020). We assessed locomotor activity, working memory, and behavioral flexibility at four time points: baseline (prior to any drug exposure), chronic exposure (14 days of drug exposure), withdrawal (2 days without drug), and finally following drug challenge (single, repeat drug exposure). Changes in cognitive performance and motor activity were determined relative to baseline, pre-drug exposure levels. To our knowledge, this is the first study to examine the cognitive effects of psychostimulant sensitization in zebrafish.

## Materials and methods

### Subjects and drugs

Thirty-six adult, AB wild-type, mixed sex (50:50 M:F) zebrafish (*Danio rerio*), aged 3 months old at the start of treatment, were used for this study. Sample size (*N* = 36 (*n* = 12/treatment group) [50:50 M:F]) was determined by power analyses based on large effect sizes (*f*^2^ > 0.3) observed in extensive previous experiments in our group using the FMP Y-maze. Housing conditions were maintained on a re-circulating system (Aquaneering Inc., San Diego, CA, USA); tank water was maintained at pH 8.4 (± 0.4), temperature 25–27 °C, on a 14-h:10-h light/dark cycle. From free-feeding (5 days post fertilization (dpf)), fish were fed on ZM fry food until adulthood when they were fed on a daily diet of live brine shrimp (maintained at the fish facility) and dried flake food (ZM Systems, UK) 3 times/day during the week and once/day at weekends. All fish used in this study were experimentally naïve. Ethical approval was granted by the University of Portsmouth Animal Welfare and Ethical Review Board and conducted in accordance to the Animals (Scientific Procedures) Act 1986. Drugs were obtained from Sigma-Aldrich (Dorset, UK). Drug solutions were made up in aquarium water at pH 8–9. Moderate doses of AMPH and NIC were based on previous studies which found these concentrations to have reinforcing effects on zebrafish, assessed in the conditioned placed preference (CPP) task (Brock et al. 2017; Kalueff 2017; Kily et al. 2008; Ninkovic and Bally-Cuif 2006; Parker et al. 2013a, b). All experiments were carried out in line with ARRIVE guidelines (NC3Rs, UK).

### FMP Y-maze

**Fig. 1:**
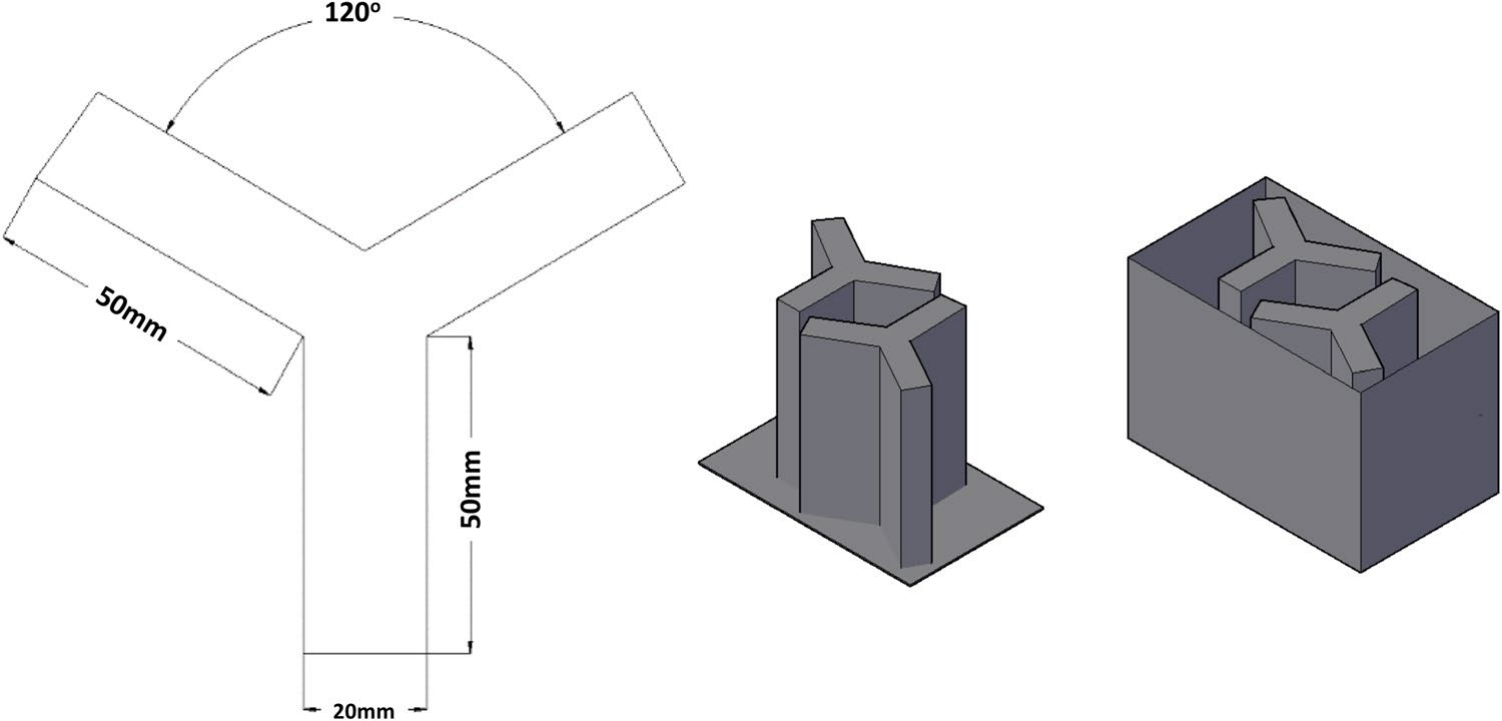
Schematic showing dimensions of individual Y-mazes (*left*), two Y-maze inserts (*middle*), and two Y-mazes inserted into a watertight tank (*right*). One zebrafish per Y-maze

The FMP Y-maze has been previously shown to be a useful method for assessing working memory, behavioral flexibility, and locomotion, and has been described in detail elsewhere (Cleal et al. 2020; Cleal and Parker 2018; Fontana et al. 2019). Briefly, fish were placed individually in white acrylic maze inserts (arms: L50 × W20 × D140 mm). Each arm of the maze was set at 120° angles. There were two inserts per tank, allowing two individuals to be tested simultaneously. The inserts were fitted into a tank containing 3L of aquarium water (Fig. 1). The FMP Y-maze comes as a standard protocol within the commercially available automated behavioral tracking unit (Zantiks AD system for adult zebrafish, Zantiks Ltd, Cambridge, UK). Fish were free to explore all arms of the maze for 1 h (h). Entries and exits from each arm were automatically recorded and logged in an excel file for the duration of the task. Arm entry data was extrapolated to obtain a time series of left and right turns, which were subsequently divided into 16 overlapping sequences of tetragrams (four consecutive turn choices, e.g. left, left, right, right [LLRR] or right, left, right, left [RLRL]; for details of analysis of tetragrams, see the “Data analysis” section below). Analyses of temporal dynamics of search patterns have demonstrated that zebrafish show flexibility in their behavior during the search parameters. Pharmacological analyses have demonstrated that alternations (LRLR, RLRL) are reduced by memory-blocking drugs in zebrafish (Cleal et al. 2020), suggesting that alternations are linked to working memory. Locomotion was measured by the total number of arm entries.

### Experimental design

**Fig. 2:**
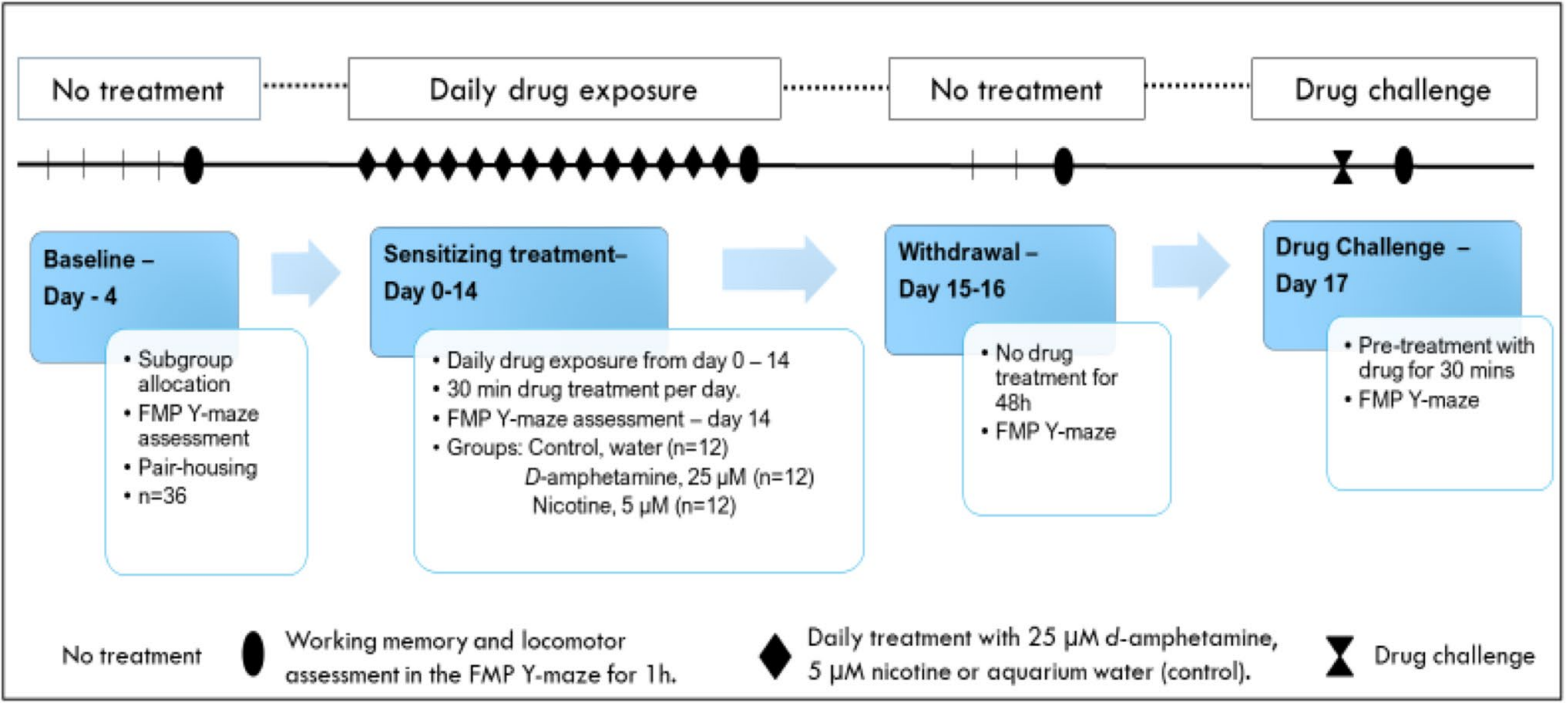
Schematic representation of working memory, cognitive flexibility, and locomotor assessments during pre-treatment phase to establish baseline performance, following 14 days of daily drug treatment (intermittent exposure), after 2 days of rest (withdrawal) and following repeat drug treatment (drug challenge) to examine AMPH and NIC sensitization

First, all fish were screened for baseline performance in the FMP Y-maze for 1 h of free exploration. Zebrafish were then allocated randomly into treatment groups (*n* = 12 AMPH [25 µM]; *n* = 12 NIC [5 µM]; *n* = 12 control) and pair-housed in treatment groups for identification purposes. Experimenters were blinded to treatment-group allocation. We pair-housed the fish 4 days prior to the start of repeated intermittent exposure to drugs in order to ensure they had habituated to the new housing system, and during this time, technical staff were blinded to treatment allocation. Acquisition of AMPH or NIC sensitization was established over three treatment phases (Fig. 2). Phase I: each fish was given 14 consecutive 30-min daily sensitizing exposures to their respective treatment. On day 14, following their final 30-min exposure, fish were immediately placed into the FMP Y-maze for 1 h of free exploration. Phase II: zebrafish had 2 days without treatment wherein they remained in their home tanks, in pair-housed conditions. After 48 h of withdrawal, zebrafish were recorded in the FMP Y-maze for 1 h. Phase III: each group received a challenge dose of their respective treatment for 30 min. Immediately following drug challenge, zebrafish were recorded in the FMP Y-maze for 1 h.

### Data analysis

All analyses were carried out using IBM SPSS Statistics (v26) and graphical representations were completed using GraphPad Prism (v8). Studentized residuals were generated from statistical models and screened for outliers. Any data point identified as an extreme value (> 3*IQR) was removed from further analysis. Previous work from our lab has identified two regular tetragram patterns (alternations [LRLR + RLRL] and repetitions [RRRR + LLLL]) that appear disproportionately regularly (compared to other sequences) throughout the duration of the trial. In order to study locomotor responses, we analysed each treatment stage based on the total number of turns completed during 1 h of exploration in the maze. LMM analysis was carried with “total turns” as the dependent variable, “treatment (AMPH, NIC, control)” and “treatment stage” (baseline, sensitizing, withdrawal, challenge) as the within-subjects factor, and “ID” as a random effect. Analysis was followed by the Bonferroni-corrected post hoc test comparing each stage to baseline.

Cognitive flexibility was based on changes in alternation strategy during trial progression. The percentage of alternations used during each 10-min time bin for six successive time bins was analysed using LMM with “alternations” as the dependent variable, “time” (6 levels, 10-min time bins), “treatment” and “treatment stage” as the within-subjects factors, “total turns” as a covariate, and “ID” as a random effect. Analysis was followed by the Bonferroni-corrected post hoc analysis comparing each time bin to every other time bin for each treatment group. Data are represented as mean + standard error of the mean for bar charts and mean ± SEM for scatter plots. Alpha values of *p* ≤ 0.05 were considered statistically significant.

## Results

### Development of sensitizing effects of AMPH and NIC on working memory and stereotypic behavior in adult zebrafish

**Fig. 3:**
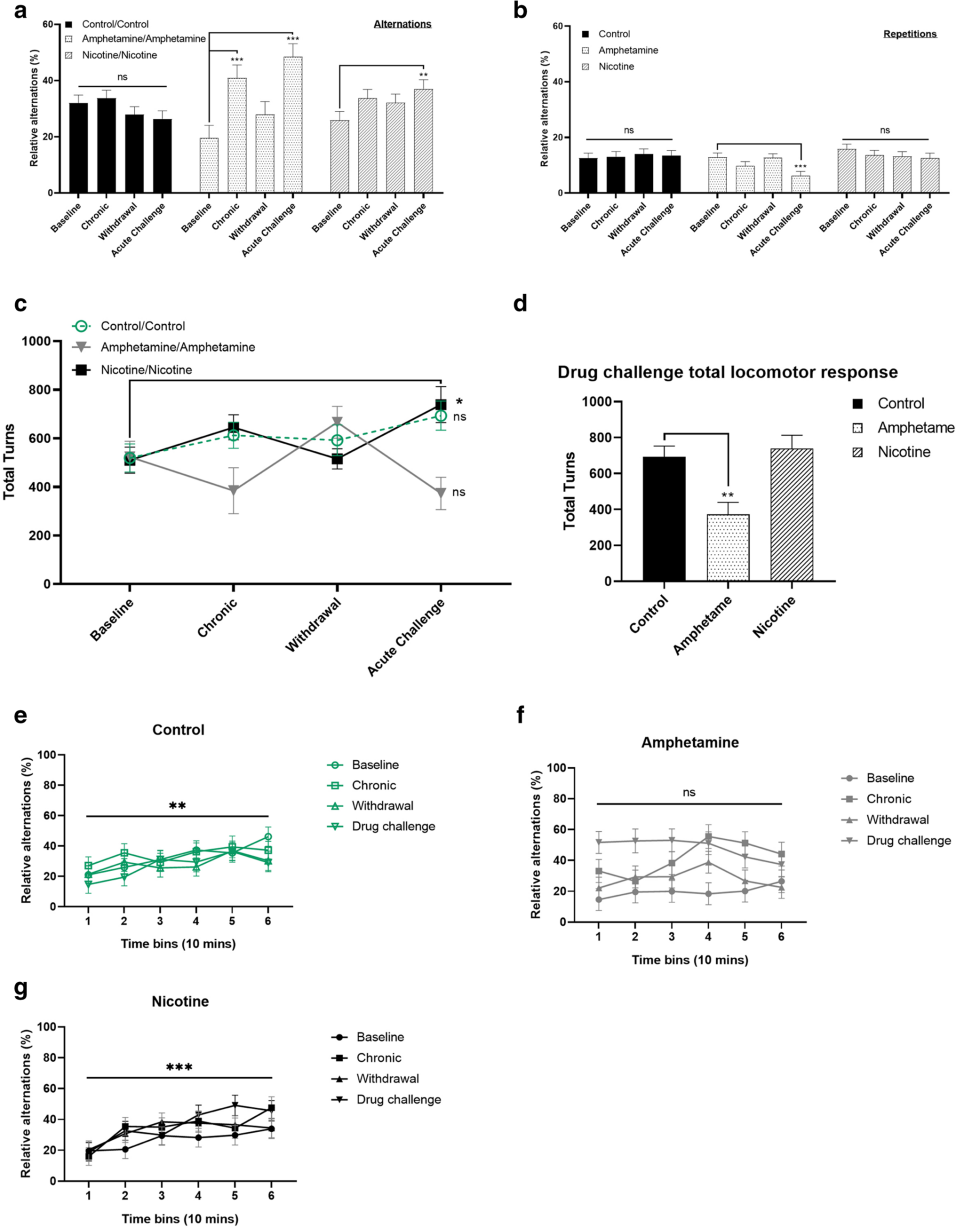
Behavioral response to repeat drug exposure in adult zebrafish treated with NIC or AMPH. Analysis of drug-induced sensitization of AMPH- and NIC-induced cognitive enhancement. Working memory and stereotypic behavior were determined during several stages of drug exposure using a repeated measure analysis of pre-treatment (*baseline*) abilities compared to cognition following 14 days of sensitization to either water (*control/control*), AMPH (*AMPH/AMPH*), or NIC (*NIC/NIC*), 2 days of withdrawal and after challenge with either water, AMPH, or NIC (*n* = 11 control, *n* = 11 AMPH, *n* = 12 NIC treated). All animals were given an acute dose of the same treatment that they were sensitized to. Data are expressed as mean + SEM of total percentage use of (A) alternations for assessment of working memory, (B) repetitions for the assessment of stereotypic behavior and mean ± SEM of (C) locomotor activity based on total turns completed during 1 h of exploration. Data were analysed using a LMM analysis followed by the Bonferroni post hoc analysis comparing each stage to baseline. (D) Analysis of total locomotor activity after repeated administration of water, AMPH, or NIC. Analysis of percentage use of alternations over time for each treatment stage per treatment group was analysed using LMM followed by the Bonferroni post hoc analysis using pairwise comparison of each 10-min time bin for a total of 6 time bins for (E) controls treated with water, (F) conditioned to AMPH followed by acute AMPH challenge, and (G) conditioned to NIC followed by acute NIC challenge. Data are expressed as mean ± SEM. *p < 0.05, **p < 0.01, ***p < 0.001; ns, not significant

Use of alternation strategy in the FMP Y-maze has been linked with changes in working memory. Adult zebrafish were assessed at pre-treatment stage, prior to drug exposure and then subsequently after 14-day intermittent exposure, withdrawal, and finally, following drug challenge, to assess sensitization effects in water-treated (*n* = 12), AMPH-treated (*n* = 12), and NIC-treated (*n* = 12) adult zebrafish. LMM showed a significant effect of treatment stage (*F*_3, 683_ = 10.23, *p* < 0.001) and a significant treatment × treatment stage interaction (*F*_3, 684_ = 6.84, *p* < 0.01). There was no effect of treatment alone (*F*_2, 30_ = 1.16, *p* = 0.33). Control fish treated with water showed no difference in alternation use between any treatment stage (*p* = 0.12). AMPH-treated fish demonstrated a significant effect of treatment stage with increased alternations after sensitization (95% CI, 12.24–30.60, *p* < 0.001), returning to near baseline levels after withdrawal (although this effect fell short of significance: 95% CI, − 0.141 to 16.75, *p* = 0.06). Drug challenge with AMPH caused alternation levels to increase significantly above baseline level (95% CI, 18.87–37.48, *p* < 0.001), demonstrating an increase in alternation use by ~ 11% after sensitization and ~ 19% following acute drug exposure. NIC has a significant effect on treatment stage (*p* = 0.03). Similarly to AMPH, NIC-treated fish showed no difference between sensitization and baseline (95% CI, − 0.814 to 16.45, *p* = 0.09) or withdrawal and baseline (95% CI, − 1.98 to 14.46, *p* = 0.21) alternation levels, but demonstrated a significant increase in alternations from ~ 25% at baseline to ~ 36% following sensitization (95% CI, 1.889–20.376,* p* = 0.01) compared to pre-treatment levels (Fig. 3A). Neither controls (*p* = 0.92) nor NIC-treated fish (*p* = 0.29) showed changes in repetition use at any treatment stage; however, AMPH-treated fish showed a significant effect of repetitions on treatment stage following drug challenge compared to baseline (95% CI, − 10.64 to − 2.67, *p* < 0.001) (Fig. 3B).

### Locomotor sensitization induced by AMPH and NIC

Locomotor activity was assessed by number of turns completed during 1 h of exploration. LMM revealed a significant effect on locomotor activity of treatment (*F*_2, 122_ = 4.47, *p* = 0.01) and treatment × treatment stage interaction (*F*_3, 122_ = 4.0, *p* < 0.01), but no main effect of treatment stage (*F*_3, 122_ = 1.15, *p* = 0.33). NIC demonstrated psychomotor sensitization following acute NIC challenge, resulting in hyperlocomotion compared to baseline (95% CI, − 4.41 to 16.13, *p* = 0.03) (Fig. 3C), while AMPH challenge caused significant hypolocomotion compared to controls (95% CI, 99.99–539.8, *p* < 0.001) (Fig. 3D).

### AMPH treatment inhibits cognitive flexibility

To assess cognitive flexibility, the percentage use of alternations was analysed as a factor of time. For each treatment group, LMM was used to examine the effect of treatment stage on alternations per time bin for six successive 10-min time bins. For controls, there was a significant effect on alternations of time (*F*_5, 208_ = 4.06, *p* < 0.01), but no main effect of treatment stage (*F*_3, 210_ = 2.12, *p* = 0.10), or treatment stage × time interaction (*F*_5, 208_ = 0.55, *p* = 0.91) (Fig. 3E). NIC caused a significant effect on alternations of time (*F*_5, 222_ = 7.01, *p* < 0.01) of treatment stage (*F*_3, 223.7_ = 2.70, *p* = 0.05), but no effect of treatment stage × time interaction (*F*_5, 222_ = 0.70, *p* = 0.79) (Fig. 3G). AMPH-treated fish showed a significant effect on alternations of treatment stage (*F*_3, 191_ = 20.91, *p* < 0.001), but no effects of time (*F*_5, 190_ = 1.42, *p* = 0.22) or a treatment stage × time interaction (*F*_5, 190_ = 1.35, *p* = 0.18). Collectively, this demonstrated a significant impairment to alter use of alternations over time in fish treated with AMPH (Fig. 3F).

## Discussion

Here, we investigated the sensitizing effects of the psychostimulants AMPH and NIC on both cognitive abilities and locomotor responses in zebrafish. Psychostimulants have performance-enhancing effects, best characterized in subjects with low baseline levels of cognitive abilities or pre-existing disorders that cause cognitive deficits, such as schizophrenia or attention deficit hyperactivity disorder (ADHD) (for reviews, see Valentine and Sofuoglu 2017; Wood et al. 2014). AMPH is therefore the front-line pharmaceutical treatment for ADHD, with many studies reporting improvements in attention and concentration (Faraone and Biederman 2002; James et al. 2001; Spencer et al. 2006). Nicotine has similarly been found to ameliorate cognitive impairment in ADHD (Levin et al. 1996, 2006; Liebrenz et al. 2014; Rezvani and Levin 2001; Wilens 2003). Although less conclusive, improvement in certain cognitive domains has also been reported in healthy subjects treated with nicotine or “clinically relevant” doses of AMPH (Bagot and Kaminer 2014; Barch and Carter 2005; Heishman et al. 2010; Jasinska et al. 2014; Mattay et al. 2000; Turner et al. 2003; Valentine and Sofuoglu 2017; Wood and Anagnostaras 2009). Here, we demonstrate that administering a moderate dose of AMPH, but not NIC, directly into the water of healthy adult zebrafish, for 14 days of intermittent, daily exposure, improved working memory compared to baseline levels. In addition, unlike AMPH, NIC did not cause degradation of cognitive flexibility, suggesting that it might have fewer unwanted negative effects compared to AMPH.

Repeat administration of psychostimulants induces behavioral sensitization when exposed to a subsequent drug challenge, and this often manifests as an enhancement of cognitive function (Berridge et al. 2012). Following repeated intermittent exposure, both AMPH- and NIC-treated adults showed cognitive sensitization, as evidenced by enhancement of performance, with alternation use increasing by ~ 11% for AMPH and ~ 8% for NIC (compared to baseline) immediately following chronic dosing. A further increase of ~ 8% and ~ 3%, respectively, was reported following the challenge dose. Decreased alternations in the FMP Y-maze have been pharmacologically characterized as a reduction of working memory, suggesting that increased alternations may be indicative of improved working memory (Cleal et al. 2020). The sensitization of certain cognitive domains, such as working memory, is likely due to altered neurotransmission in ascending DA pathways from the striatum (Berke and Hyman 2000). Previous studies have shown that after chronic administration of a psychostimulant, subsequent doses cause increased DA release (Herman and Roberto 2015). The duration of this increased DA response can be several weeks or, as demonstrated here, can be evoked after 48 h of withdrawal from sensitization treatment.

AMPH and NIC increase striatal DA release (Krause et al. 2002), which can enhance striatum-dependent tasks, such as those requiring working memory and memory consolidation (Brown et al. 2000; Landau et al. 2009), and subsequently potentiate performance of a previously established pattern of behaviors, in this case, the increased use of alternations. Elevated striatal DA increases the use of “habits”, blocking the extinction of established behaviors following devaluation of the goal (Berke and Hyman 2000). This may provide a possible mechanism for the persistent use of alternations, at the expense of other strategies, after AMPH-induced sensitization. This response strategy may also represent persistent behavioral changes that are associated with drug addiction, in which the persistent seeking and taking of drugs of abuse come at the expense of other normal and necessary behaviors. The fact that this habitual use of alternations, in favour of other search strategies, is only evident in AMPH-treated fish could be suggestive of the different mechanisms of increasing extracellular DA through dopaminergic or cholinergic pathways. Alternatively, the differences we observed between AMPH and NIC could be a result of the concentration used, and thus future studies should examine the sensitizing effects of a range of drug concentrations.

Psychomotor stimulants are known for their motor activity–altering effects (Cunningham et al. 1997). Repeated, intermittent exposure to psychostimulants causes progressive enhancement of locomotor activity with subsequent challenge exposures (Adriani et al. 2006; Kuczenski and Segal 2002; Peleg-Raibstein et al. 2009; Pisera-Fuster et al. 2019). Here, NIC treatment caused sensitized hyperlocomotion, whereas AMPH-treated fish demonstrated hypolocomotion. Treatment with AMPH has been reported to have a paradoxical effect of cognitive enhancing attentional increases and behavioral-calming reductions of hyperactivity. Many studies have shown that these effects are evident not only in patients diagnosed with ADHD but also in healthy subjects (Bagot and Kaminer 2014; Holze et al. 2020; Roberts et al. 2020). Human studies with healthy participants have shown that dosing with AMPH can cause an overall decrease in activity levels in healthy boys and adults and similar decreases in hyperactive boys. At low–moderate concentrations of AMPH (as used here), increased hypoactivity is correlated with improved memory performance in cognitive tasks (Rapoport et al. 1980; Spencer et al. 2015). Here, we demonstrate improved cognitive performance corresponding to hypolocomotion in zebrafish.

NIC has regularly been reported as causing hyperlocomotion. Progressive long-lasting increases in locomotor and DA-activating effects are generally associated with moderate exposure regimens, whereas high doses of NIC for prolonged periods are more closely associated with signs of withdrawal and typically do not demonstrate locomotor sensitization (Pisera-Fuster et al. 2019). Several studies have shown that a single low dose of NIC is ineffective at inducing locomotor or dopaminergic activation; however, daily low doses can induce locomotor sensitization after days of withdrawal from drug treatment (Fennell et al. 2020; Pisera-Fuster et al. 2019). These conclusions are in line with our findings here, demonstrating no differences in locomotor effects following intermittent dosing; however, after just 2 days of withdrawal, drug challenge resulted in psychomotor sensitization, demonstrated by a significant rise in motor activity from baseline to repeat drug challenge.

The goal of exploring the FMP Y-maze could be perceived as information seeking or foraging behavior. As fish become familiar with and continue to explore the test arena, they are continuously updating their knowledge of the environment and this can subsequently be used to alter search strategies. In the FMP Y-maze, the absence of reward during the 1 h of exploration has been shown to instigate a change in alternation use in healthy adult zebrafish. Many studies have investigated the relationship between DA agonists, such as AMPH and methylphenidate (MPH), and the relationship between different cognitive domains such as working memory, attention, and cognitive flexibility (Bagot and Kaminer 2014). The inverted U–shaped response to psychostimulants has often been reported to result in the simultaneous enhancement and impairment of multiple cognitive functions (Wood et al. 2014). Studies investigating effects of psychostimulants on cognitive function have reported that high levels of sustained attention correlate with lowered levels of cognitive flexibility, assessed using set shifting tasks (Berridge et al. 2012).

The PFC has been found to be a key region for the regulation of cognitive flexibility (Rougier et al. 2005). Both high prefrontal D1 receptor stimulation and low prefrontal D1 receptor stimulation have been linked with impaired flexibility responding in humans, using the Wisconsin Card Sorting Task (WCST) (Klanker et al. 2013; Takahashi et al. 2008, 2012). In a previous study conducted by our lab, we administered a serial dilution of acute doses of the D1-like antagonist SCH-23390 prior to testing in the FMP Y-maze. We found that SCH-23390 severely impaired cognitive flexibility in the FMP Y-maze at the highest dose (1.5 mg/L), but not at the lower doses (0.5–1.0 mg/L) which still maintained an effect of time on alternations. Additionally, all doses of SCH-23390 caused a significant reduction in overall use of alternations, hypolocomotion, and, at the highest dose, an increase in stereotypic behavior, shown by a significant increase in repetitions (Cleal et al. 2020). These findings, in conjunction with the present work, demonstrate a complex role for D1 in modulating cognitive flexibility. Further work is required to disentangle the relationship between DA receptor activation and extracellular levels of DA to fully understanding the mechanisms involved in maintaining cognitive flexibility and other cognitive functions.

## Conclusion

We investigated the sensitizing effects of AMPH and NIC on working memory, locomotion, and cognitive flexibility. We demonstrated that non-contingent exposure to the psychostimulant AMPH, but not NIC, improved working memory performance in the FMP Y-maze after 14 days of intermittent dosing, returning to basal levels after 2 days of abstinence. Subsequent challenge resulted in sensitization of both drugs, resulting in greater use of alternations, demonstrating an increase in the preference for this exploration strategy. In AMPH, this increase in alternation use was accompanied by a decrease in the use of other strategies, demonstrating a focused increase, bordering on stereotypic behavior. Only NIC demonstrated increased locomotor sensitization compared to pre-drug exposure levels. AMPH caused significant hypolocomotion during challenge. Additionally, our findings suggest a role for dopaminergic, but not cholinergic, overactivation in the inhibition of cognitive flexibility. Although the protocol for psychostimulant-induced motor sensitization is substantially less cumbersome to establish than other models of addiction, it is not a precise homologue for psychostimulant addiction in humans (Berke and Hyman 2000). Understanding drug-induced changes in neurotransmitter systems and neural plasticity by studying cognitive sensitization may provide a useful model with high face validity. These results demonstrate that previous exposure of fish to AMPH or NIC can enhance cognitive performance, but this may come at a cost, with improved performance in one cognitive domain potentially resulting in disruption in another domain. This may demonstrate the first signs of drug-induced maladaptive activities. The distinction between cognitive enhancement and inhibition of normal behaviors may be dependent on cognitive flexibility, the loss of which has been strongly linked with substance abuse disorders which are often characterized by deficits in cognitive flexibility and may underlie the persistence of harmful, drug seeking and taking behaviors.

Finally, this paper is the first to demonstrate robust test–retest reliability for the FMP Y-maze. In previous studies, we have only looked at single exposures of animals to the maze. Our control animals showed no significant changes in total turns, alternations, repetitions, or general search patterns in the maze between their four test periods, suggesting that the FMP Y-maze can be used longitudinally to examine changes in memory or behavioral flexibility over time.
